# Idiopathic Inflammatory Myopathies: A Review of the Classification and Impact of Pathogenesis

**DOI:** 10.3390/ijms18051084

**Published:** 2017-05-18

**Authors:** Dana E. Mandel, Charles J. Malemud, Ali D. Askari

**Affiliations:** Department of Medicine, Division of Rheumatic Diseases, Case Western Reserve University School of Medicine and University Hospitals Cleveland Medical Center, Cleveland, OH 44106-5076, USA; cjm4@cwru.edu (C.J.M.); ali.askari@uhhospitals.org (A.D.A.)

**Keywords:** idiopathic, inflammation, muscle diseases

## Abstract

Idiopathic inflammatory myopathies (IIMs) are a group of autoimmune muscle diseases with significant morbidity and mortality. This review details and updates the pathogenesis and emerging importance of myositis-specific antibodies in the development of IIMs. An increase in the understanding of how these myositis-specific antibodies play a role in IIMs has led to the further categorization of IIMs from the traditional polymyositis versus dermatomyositis, to additional subcategories of IIMs such as necrotizing autoimmune myositis (NAM). The diagnosis of IIMs, including manual muscle testing, laboratory studies, and non-invasive imaging have become important in classifying IIM subtypes and for identifying disease severity. Treatment has evolved from an era where glucocorticoid therapy was the only option to a time now that includes traditional steroid-sparing agents along with immunoglobulin therapy and biologics, such as rituximab.

## 1. Introduction

Idiopathic inflammatory myopathies (IIMs) are a group of autoimmune muscle diseases with significant morbidity and mortality if not properly addressed during a clinical evaluation early on in the disease course. This group of myopathies has long been classified based on clinical criteria created by Bohan and Peter in 1975. However, continued research has demonstrated the utility of increased subclassifications of myopathies, often based on the presence of antibodies, which has shed further light on the pathophysiology and treatment options for inflammatory myopathies [1].

IIMs are estimated to occur in 4.27–7.89 cases/100,000 individuals per year [1]. IIMs, excluding inclusion body myositis, typically present with proximal muscle weakness and may progress to systemic involvement of organ systems, such as the lung and the heart, with significant morbidity. Schiopu et al. [2] studied 160 patients with the diagnosis of polymyositis/dermatomyositis and the effect of corticosteroids, methotrexate, and azathioprine on the course of the disease. The results of this study demonstrated a 10-year survival rate of 62% [1,2]. Increased mortality was noted in the patients who received intravenous glucocorticoid therapy which was postulated to be due to increased severity at disease presentation; methotrexate and azathioprine treatment groups did not show a significant difference in mortality [2]. Additional attempts have been made by multiple investigative groups to further classify the pathophysiology of IIMs in order to better diagnose and predict which groups of patients have an increased likelihood for the development of malignancy, interstitial lung disease (ILD), and other co-morbidities associated with IIMs.

In that respect, the treatment of inflammatory myopathy is changing, once only consisting of treatment with high-dose glucocorticoids to now include traditional immunosuppressive steroid-sparing immunotherapy as well as immunoglobulin and biologic agents, such as rituximab infusion. The exciting science associated with uncovering the pathophysiological underpinnings of IIMs has become more intricate, and treatments have expanded over years of research, particularly with the findings of myositis-specific antibodies.

## 2. Clinical Presentations

An extensive review of the current published literature on IIMs has increasingly divided the disease into the following five subcategories: polymyositis, dermatomyositis, necrotizing autoimmune myopathy, sporadic inclusion body myositis, and anti-synthetase syndrome, each of which have unique clinical presentations. Applying these five subcategories, the typical clinical presentations are presented below.

### 2.1. Dermatomyositis

Dermatomyositis classically presents with proximal symmetric muscle weakness and associated characteristic rashes. The characteristic skin rashes include the shawl sign or V sign, Gottron papules, and heliotrope rashes over the eyelids with associated periorbital edema [1,3]. Creatine kinase (CK) levels may be elevated up to 50 times the upper limit of normal in the subacute active phase. However, patients with dermatomyositis may also present with characteristic rashes, but lacking muscle weakness, which is known as amyopathic dermatomyositis. Patients may also present with muscle weakness and typical muscle biopsy pathology but lack rashes, which is referred to as dermatomyositis *sine* dermatitis [3,4]. Approximately 15% of dermatomyositis patients diagnosed after the age of 40 have, or will develop, a malignancy within 3–5 years following diagnosis. The most common malignancies associated with dermatomyositis include colorectal, ovarian, lung, pancreatic, and stomach cancers [1,4].

### 2.2. Anti-Synthetase Syndrome

Separating itself from the other subcategories, anti-synthetase syndrome consists of typical symmetric proximal muscle weakness with biopsy findings consistent with dermatomyositis, but with an associated positive anti-Jo1 myositis-specific antibody in 75% of patients. Anti-synthetase syndrome typically presents with these findings, along with arthritis, Raynaud’s phenomenon, fever, and “mechanic’s hands”, with 70% of patients developing ILD. This spectrum of clinical presentation has led to this separate and unique subcategory of IIMs [4].

### 2.3. Necrotizing Autoimmune Myositis

Necrotizing autoimmune myositis (NAM) is a subcategory estimated to account for 20% of IIM cases, which, prior to the increased importance of histopathology, were likely classified as polymyositis [3]. NAM may present acutely or subacutely with a significant proximal symmetric weakness, which is often severe and debilitating at initial presentation. Serum CK is often elevated compared to polymyositis/dermatomyositis with levels greater than 50 times the upper limit of normal. Myositis-specific antibodies, anti-signal recognition particle (anti-SRP), and 3-hydroxy-3-methylglutaryl coenzyme A reductase antibodies (anti-HMGCR) are specific for NAM. Initially theorized to occur secondary to statin exposure, the recent published literature notes that patients may not have had exposure to statins but may develop anti-HMGCR antibody with exposure from other sources [4].

### 2.4. Polymyositis

Polymyositis is increasingly being recognized as a bucket term for IIMs that do not have the specific criteria for placement into the other four subcategories or which are negative for myositis-specific antibody. Patients typically present in a manner similar to dermatomyositis with subacute proximal symmetric muscle weakness; however, polymyositis patients are without characteristic rashes of dermatomyositis and have yielded different findings with respect to muscle histopathology, as demonstrated in Table 1. Serum CK levels may be elevated up to 50 times the upper limit of normal in the subacute active phase [1,3,4,5].

### 2.5. Sporadic Inclusion Body Myositis

Inclusion body myositis may present in a manner similar to polymyositis with respect to muscle biopsy pathology in early disease, as CD8^+^ T-cells are similarly predominant on histopathology. Staining for cytochrome-oxidase, p62, and congophilic amyloid deposits while also assessing the presence of vacuoles can differentiate inclusion body myositis from polymyositis [1,4,6]. However, it is noted that these characteristic histopathologic findings for inclusion body myositis may not be present in early disease activity, which is why initial histologic exam may be more consistent with the findings seen in polymyositis [6]. A closer clinical assessment can lead to a differentiation which is important as treatment options for inclusion body myositis lack compared to treatment of the other subtypes of IIMs [7]. Inclusion body myositis predominately initially affects the distal muscles, including wrist extension, forearm motion, and fine motor activity of the hands, with an associated involvement of the quadriceps. Although atypical for other IIMs, it may also affect facial and axial muscles. Dysphagia is also seen. This disease process is more insidious with a noted slowly progressive onset occurring in patients over the age of 50. The disease predominantly affects men. Serum CK may be elevated up to 10 times the upper limit of normal [3,8].

### 2.6. Diagnosis

IIMs combined with further defining a patient’s subcategory are based on an interpretation of clinical and diagnostic tests. For example, the physical exam findings, including manual muscle testing, are particularly important in discriminating between inclusion body myositis and other IIM subcategories [8]. Symmetric proximal muscle weakness is the predominant feature. Neck flexor weakness, as well as dysphagia in severe disease, may be present [1]. Laboratory testing should be used for disease diagnosis and for monitoring responses to treatments along with monitoring for disease flares. Serum CK is particularly important for monitoring disease progression; however, importantly, the initial level of CK elevation can vary within IIM subcategories as noted above [4]. Similarly, myoglobin and aldolase levels can be elevated. These can also be used as a monitor of disease progression. Serum glutamic oxaloacetic transaminase (SGOT) and serum glutamic pyruvic transaminase (SGPT) may be elevated secondary to both liver disease and muscle breakdown. This can result in difficulty in monitoring for medication side-effects, particularly in patients treated with methotrexate (MTX), where the gamma-glutamyl transferase level may be needed to separate SGOT/SGPT elevation due to muscle breakdown versus hepatotoxicity [7]. Thyroid function tests should be performed as myositis can occur in patients with thyroid abnormalities [1,4].

Electromyography (EMG) and nerve conduction studies have been used to separate neuropathic versus myopathic causes of proximal muscle weakness prior to biopsy [4]. EMG, which is typically performed unilaterally on the upper and lower extremity in IIMs, demonstrates a characteristic short duration, low amplitude polyphasic units on voluntary activation with increased spontaneous fibrillations and positive sharp waves [4]. Despite the characteristic involvement of proximal limb muscles involvement, testing of paraspinal muscles has been performed in patients with IIMs, particularly when patients present with truncal weakness [1].

Magnetic resonance imaging (MRI) is gaining increased importance in diagnosing IIMs and for guiding treatment options. Patients with contraindication to EMG may be considered for MRI evaluation to assess edema, inflammation, adipose infiltration, and atrophy [4]. MRI can also guide muscle biopsy location for increased yield [4]. Day et al. reviewed the use of MRI in IIMs in 2016 [9]. MRI imaging, particularly fat suppressed images, can be useful in identifying muscle regions with active inflammation versus regions of fat infiltration. Appropriately, the review noted that many etiologies also associated with elevated CK and may cause inflammatory changes on MRI of muscle tissue; thus, muscle biopsy remains the gold standard in diagnosis [9]. However, MRI can be used to increase the yield of muscle biopsy by directing the biopsy to be performed in a location with active inflammation versus region(s) with chronic changes or fat infiltration, which may have non-diagnostic histopathology [9]. The review also noted that MRI had a sensitivity of 89–100% when using short-T1 inversion recovery (STIR) images and the specificity of MRI imaging was 80–88% [9]. Although there is no clear consensus on using MRI imaging for monitoring disease activity, MRI may be considered as a tool for differentiating active versus chronic disease activity [9].

In patients with cardiac muscle involvement, echocardiogram may be used in screening and evaluation [5,10]. Patients with anti-Jo1 positivity or symptoms and where concern exists for lung involvement should undergo pulmonary function testing and/or high resolution CT imaging for evaluation of ILD [5].

Muscle biopsies do remain the gold standard for diagnosis and differentiation of subcategories of IIMs. Thus, in a retrospective study of 27 patients the results showed that, for a definite diagnosis of IIMs, muscle biopsy from two sites was required 70% of the time, whereas one site yielded a definitive diagnosis only 30% of the time [1]. The typical muscle pathology findings on biopsy of IIM patients are shown in Table 1.

## 3. Overview of the Pathophysiology of Inflammatory Myopathies

The pathophysiology of inflammatory myopathies varies within subcategories. Multiple attempts have been made at reclassifying IIMs since the classification of Bohan and Peter in 1975. Their work classified these patients as either polymyositis or dermatomyositis, with patients that lack rashes being labeled as polymyositis [1,11,12]. However, in 2003, Dalakas & Hohfeld [13] proposed the following definitions based on muscle biopsy pathology and immunology for characterizing IIMs: (i) definite polymyositis demonstrates inflammation with CD8^+^ T-cells/MHC-1 complex and no vacuoles; (ii) probable polymyositis demonstrates inflammation with MHC-1 but without CD8^+^ T-cells and no vacuoles; (iii) definite dermatomyositis demonstrates inflammation with perifascicular, perimysial, or perivascular infiltrates and perifascicular atrophy, together with the presence of rashes on clinical exam; and (iv) probable dermatomyositis demonstrates the typical muscle biopsy pattern noted for definite dermatomyositis but without the presence of rashes on clinical exam [1,5,13]. Furthermore, in 2003, the Myositis Study Group and the European Neuromuscular Centre workshop also sought to provide a new classification system that specified criteria for definite versus probable polymyositis, definite versus probable dermatomyositis, amyopathic dermatomyositis, probable dermatomyositis *sine* dermatitis, nonspecific myositis, and lastly immune-mediated necrotizing myopathy [5,14].

Despite these advances in classifying inflammatory myopathies, ongoing research has provided advances yet confounding theories on the exact pathophysiologic mechanism whereby IIMs are triggered and how the disease pathways vary between subcategories. A viral trigger has been theorized in a manner similar to other autoimmune diseases. In addition, associated genetic and HLA alleles are also being studied, noting associations such as DRB1*0301 and anti-Jo1 positivity as well as DRB1*1101 and 3-hydroxy-3-methylglutaryl coenzyme A reductase antibody (anti-HMGCR) positivity [1,4,15]. Thus, in patients with a diagnosis of dermatomyositis, a review of current pathophysiology supports a theory of antigen activation of the C5b-9 macrophage-activating complex (MAC). Once activated, MAC is deposited on the surface of endothelial cells, and is recognized as the antigenic target, which leads to necrosis and ultimately to capillary ischemia. This event further causes the characteristic perifascicular atrophy found by muscle biopsy. Activated MAC also trigger the release of proinflammatory cytokines leading to B-cell, CD4^+^ T-cell, and plasmacytoid dendritic cell infiltration [1,4,15]. As is the case with other autoimmune processes, plasmacytoid dendritic cells are associated with increased interferon-Type 1 production with levels of interferon-Type 1 noted to be increased in the serum of patients with dermatomyositis, which also correlates with elevated disease activity [16,17]. In patients with polymyositis and inclusion body myositis, an antigen-driven response has been shown to result in CD8^+^ T-cells infiltrating otherwise healthy muscle cells that express major histocompatibility complex-1 (MHC-1), atypical for muscle cells. In that regard, CD8^+^ T-cells release perforin and granzyme B, leading to myonecrosis and ultimately to damage of the endomysium. In all of these three subcategories, increased T_H_17 production and downstream proinflammatory cytokine release, exemplified by IL-1, IL-6, IL-15, result in a proinflammatory environment [1,4].

Furthermore, the importance of myositis-specific antibodies (MSAs) patterns in the development and classification of these diseases has emerged (Table 2). Auto-antibodies directed against nuclear RNAs have been demonstrated to occur in approximately 60% of IIM patients yet the specificity of these auto-antibodies is unclear and is still being evaluated [4]. Importantly, these autoantibody associations are being used to predict the extent of increased relative risk for myositis-associated lung disease, such as ILD, malignancy, and other comorbidities associated with IIMs.

## 4. Pathophysiological Significance of Myositis-Specific Auto-Antibodies

A long-standing debate continues to persist as to the extent to which the presence of these various IIMs autoantibodies represent accurate biomarkers for recognizing IIM subcategories or may actually have a pathogenic role in the disease process. This debate has been further complicated by recent findings indicating that autoantibodies directed towards aminoacyl tRNA synthetases may actually be associated with ILD without any evidence of myositis [18]. However, the utility of several of the autoantibodies shown in Table 2 for discriminating between various sub-categories is well-founded. For example, as indicated, anti-Mi-2 can be considered the marker for classical dermatomyositis as well as its presence being critical for predicting a good response of dermatomyositis patients to steroids and therefore a good prognosis. Conversely, anti-SRP positivity appears to be specific for polymyositis and treatment resistance. Anti-PL-7/PL-12 antibody was found to be associated with early and severe ILD as well as gastrointestinal problems [19], whereas patients with myositis that were positive for anti-Jo-1 and anti-PL-7 did not significantly differ with respect to the severity of ILD [20]. However, the ratio of lymphocytes in bronchoalveolar lavage fluid was higher in Jo-1 positive compared to PL-7-positive patients, suggesting differences in pulmonary complications between the two groups of ILD patients. Pericarditis also appears to be a comorbidity associated with anti-PL-7-positive myositis, ILD and arthritis patients [21]. As indicated, antibodies to TIF-1-γ/α (also known as p155/140) are frequently found in association with malignancy and anti-MDA-5 with progressive ILD (Table 2). New dermatomyositis-specific auto-antibodies have also recently been described and include, anti-small ubiquitin-like modifier-1 (SUMO-1) activating enzyme [18], but the significance of this antibody will require additional study going forward.

The consistent finding that these autoantibodies are directed against ubiquitously expressed autoantigens raises the question as to which T-cell reactivity is responsible for directing B-cell production of the autoantibodies in IIMs [22]. In that regard, the sera of patients with IIMs and an activated interferon Type-1 pathway contain elevated levels of B-cell activating factor compared to other IIM subtypes. Of note, a newly prominent T-cell subset identified in IIMs, namely, CD244^+^ (CD28null) may provide a link between altered adaptive immunity and IIM pathology in that CD244^+^-T-cells may be cytotoxic to CD4^+^ and CD8^+^ T-cells [17]. Furthermore, the CD244^+^ T-cells appear to be “apoptosis-resistant” [17]. Interestingly, the poor outcome in myositis patients after immunosuppressive therapy has now been “linked” to the persistence of these cells in muscle and therefore may also be the contributing factor to treatment-resistance as well, although the relative loss of T-regulatory cells could also be contributing to this clinical response [23].

## 5. Therapies

Multiple immunosuppressant agents have been tried as steroid-sparing agents in the treatment of IIMs. Inclusion body myositis is an exception from the other IIMs as it has not been shown to respond to immunosuppressant agents, including glucocorticoids. In fact, inclusion body myositis has only one indicated pharmaceutical option at this time, intravenous immunoglobulin G, discussed below [24]. The remaining pharmaceuticals discussed are options for treatment of IIM subcategories, excluding inclusion body myositis.

Glucocorticoid therapy remains the primary first-line treatment for IIMs. Patients with mild to moderate disease activity defined on clinical exam findings listed previously are recommended to receive 1 mg/kg/day, not to exceed 80 mg daily. In patients with rapidly progressive or severe disease defined as clinical exam findings such as those patients with dysphagia and/or ILD and/or severe motor weakness, pulse dose steroid therapy at 500–1000 mg daily followed by 1 mg/kg/day is employed [7,25,26]. Patients are often maintained on steroid therapy for at least 9–12 months with a slow taper beginning after 4–8 weeks of treatment if improvements in the serum CK level or muscle strength are seen. Note that 50% of patients may fail to completely respond, which should prompt reevaluation of the diagnosis or a search for secondary causes of myopathy, such as that which may be steroid-induced [7,25,26,27].

MTX along with azathioprine (AZA) are considered first-line agents in what is known as steroid-sparing therapy [7]. MTX employed at a dose of up to 25 mg/week can be initiated. However, caution must be advised in patients with ILD due to the risk of MTX-induced pulmonary toxicity [7]. Elevated serum SGOT/SGPT levels resulting from muscle breakdown may also contribute to difficulty in monitoring for signs of MTX toxicity. AZA is often preferred in patients with ILD, starting at a dose of 50 mg/day with increasing to a goal of 1.5 mg/kg/day. However, contradictory results have been reported in studies comparing MTX versus AZA in regard to survival benefit [3,24].

Intravenous immunoglobulin G (IVIG) therapy is increasingly used in IIM patients with severe or rapidly progressive disease who lack tolerance or show a poor response to glucocorticoid therapy. IVIG is one of the only treatments indicated for use in inclusion body myositis, specifically in patients suffering from oropharyngeal dysfunction [24,28]. Multiple mechanisms of action have been reported for IVIG including disruption of the *F*_c_ region causing interference with cytokine and complement activity [28]. IVIG therapy is generally performed by infusion at a dose of 2 gm/kg/month [24]. Dalakas et al. [7] conducted small population studies demonstrating efficacy of IVIG in a double-blind, controlled trial of IVIG therapy in 15 patients with refractory dermatomyositis. Limitations of IVIG therapy include time, as it is often infused over a 5 day period as well as cost. Due to these considerations, Cherin et al. [29] performed a retrospective review studying the use of subcutaneous immunoglobulin use in inflammatory myositis. Nineteen patients (7 with polymyositis, 7 with inclusion body myositis, 2 with dermatomyositis, and 3 with connective tissue disease related-IIMs) received subcutaneous IVIG. Results showed that 10/14 patients demonstrated a small increase in muscle strength score (73.6 ± 9.2 (mean ± SD) out a possible score of 88 at baseline and 76.0 ± 13 out of 88 at endpoint). The muscle disability scale (MDS) was assessed on 11 patients at baseline with a reported statistically significant decrease of 2.5 points, but the mean ± SD of the MDS at baseline of 17.8 ± 18.9 minimally decreased to 16.3 ± 21.3 at endpoint [29].

Rituximab is a monoclonal antibody directed against CD-20 on the surface of pre-B-cells and has also been increasingly used in cases of IIMs refractory to first-line therapies [24]. The Rituximab in Myositis (RIM) trial performed in 2013 by Oddis et al. [30] studied 195 patients with the following subcategories: 75 polymyositis, 72 dermatomyositis, and 38 juvenile dermatomyositis, all of whom had refractory disease despite first-line therapy with glucocorticoids and at least one steroid-sparing agent. No differences were seen between those treated at baseline with rituximab versus those initiated with rituximab at 8 weeks. However, 83% of patients of the 195 patients with refractory disease enrolled in the trial did meet the predefined definition of improvement at 20 weeks with noted decreased steroid requirements as well (20.8 mg daily at baseline compared to 14.4 mg daily at trial end) [7,24,30].

Various immunosuppressant agents has been tried in the treatment of IIMs to induce steroid-sparing. For example, mycophenolate mofetil (MPF) has been reported to be employed in multiple case series. Moreover, MPF is favored for use in patients with myositis-associated ILD, in patients with connective tissue disease-associated ILD, or those with an overlap syndrome [7,24]. Cyclophosphamide has also been used in patients with overlap syndrome. Case reports showed clinical improvement with decreased steroid requirements [24]. Similarly, tacrolimus and cyclosporine have been tried due to the presence of activated CD8^+^ T-cell in myositis-associated ILD since both drugs act to block T-lymphocyte proliferation and activation [7,24]. Although case studies have demonstrated improvement with these agents [7,24], no blinded or controlled trials have yet been performed.

Case reports have detailed the small number of IIM patients treated with either tocilizumab (anti-IL-6 receptor), tofacitinib (JAK3-selective small molecule inhibitor), or anakinra (anti-IL-1 receptor antagonist), and have reported clinical improvement. Although these studies were performed on small patient populations, limiting their extrapolation to the general population of IIM patients [1,24,31]. In addition, research demonstrating upregulation of the co-stimulatory molecules CD28 and CTLA-4 in polymyositis/dermatomyositis has prompted studies of abatacept in the treatment of IIMs [32]. Thus, case reports have demonstrated a positive clinical effect in refractory myositis patients. Importantly, the ARTEMIS trial studied the use of abatacept in patients with drug-resistant dermatomyositis or polymyositis who had received glucocorticoid therapy and at least one immunosuppressant agent for at least 3 months. Patients were randomized to receive abatacept at trial enrollment or 3 months after enrollment. After 6 months of treatment, 8 of 17 patients (47%) were considered to have shown a clinically significant improvement in muscle strength and quality of life [32]. Vitamin D therapy has also been explored. Rasheed et al. reported on a case of proximal muscle myopathy with clinical features consistent with IIMs. Deltoid muscle biopsy demonstrated non-specific muscle atrophy, and it was concluded that the patient’s symptoms were due to vitamin D deficiency [33]. In that regard, Scolletta et al. reviewed the use of vitamin D receptor agonists and their ability to block CXCL10, a chemokine found to be elevated in IIMs [28,34]. The review does note that the amount of vitamin D required to block CXCL10 is associated with risk of hypercalcemia, and further research needs to be conducted in this area [34].

TNF-α is found to be increased in IIM muscle tissue, so TNF alpha inhibition was considered as a therapeutic option for IIMs. Reports of TNF-α blockade as a treatment for IIMs have not panned out at this time and new cases of dermatomyositis/polymyositis have been reported after administration of TNF-α therapy [28].

## 6. Conclusions and Future Prospects

IIMs have significant morbidity and mortality, leading to long-term disability and potential death. Increasing knowledge is being gained on myositis-specific antibodies and their role in IIMs. The presence of these auto-antibodies are now being used with increasing frequency to predict manifestations of IIMs beside the typical muscle weakness, such as ILD and the possibility of malignancy. Moreover, interferon type-1 production is increasingly being suspected as a causative insult in inflammatory myositis, particularly dermatomyositis. With an increasing knowledge regarding the pathogenesis of IIMs, multiple immunologic agents are being tried in cases of resistant IIMs. Thus, these pathways are being targeted based on the known pathogenesis of the disease. It is hopeful that this knowledge will lower the still high mortality associated with IIMs.

## Figures and Tables

**Table 1:** Idiopathic inflammatory myopathies subcategories and classical muscle biopsy pathology findings [1,4]. The presence of vacuoles are pathognomonic for inclusion body myositis [1,4]. Idiopathic inflammatory myositis (IIM); Major histocompatibility complex (MHC).

IIMs Sub-Category	Muscle Pathology	Vacuole Formation
Polymyositis	CD8^+^ T-cells; MHC-1 antigen expression	No
Dermatomyositis	Perivascular; perimysial; perifascicular inflammation; +/− necrotic fibers; perifascicular atrophy and decreased capillaries; macrophages, B-cells and CD4^+^ T-cells	No
Autoimmune necrotizing myositis	Necrotic fibers with macrophages; absence of CD8^+^ T-cells; complement deposition may be present	No
Sporadic inclusion body myositis	CD8^+^ T-cells; cytochrome-oxidase negative; congophilic amyloid deposits	Yes

**Table 2:** Myositis specific antibodies and disease associations [2,4,8]. The specificity of autoantibodies on the associated disease processes is unknown as is their role in the pathogenicity of IIMs.

Myositis-Specific Auto-Antibodies	Disease Association(s)
Anti-Aminoacyl-tRNA (e.g., Anti-Jo1, Anti-PL-7, Anti-PL-12 (anti-alanyl-tRNA synthase))	Anti-Synthetase syndrome; ILD; gastrointestinal complications
Anti-HMGCR (3-Hydroxy-3-Methylglutaryl Coenzyme A Reductase Antibodies)	Necrotizing Autoimmune Myositis
Anti-Signal Recognition Particle (SRP)	Necrotizing Autoimmune Myositis/Polymyositis
Anti-Melanoma Differentiation-Associated Protein-5 (MDA-5)	Amyopathic Dermatomyositis; rapidly progressive ILD
Anti-Mi-2 (chromodomain-helicase-DNA binding protein 4)	Dermatomyositis with typical skin lesions
Anti-Cytosolic 5’-Nucleotidase 1A (cN1A)	Inclusion Body Myositis
Anti-Transcriptional Intermediary Factor 1-γ (TIF-1-γ/α)	Malignancy-associated Dermatomyositis
Anti-Nuclear Matrix Protein-2 (NXP-2)	Malignancy-associated Dermatomyositis; Juvenile-Dermatomyositis with calcinosis
Anti-Four & a Half Limb Domain-1 (FHL-1)	Myositis with severe muscle atrophy and dysphagia but without lung or joint involvement
